# Knockdown of NIK and IKKβ-Binding Protein (NIBP) Reduces Colorectal Cancer Metastasis through Down-Regulation of the Canonical NF-κΒ Signaling Pathway and Suppression of MAPK Signaling Mediated through ERK and JNK

**DOI:** 10.1371/journal.pone.0170595

**Published:** 2017-01-26

**Authors:** Mengbin Qin, Jinxiu Zhang, Chunyan Xu, Peng Peng, Lin Tan, Shiquan Liu, Jiean Huang

**Affiliations:** Department of Gastroenterology, The First Affiliated Hospital, Guangxi Medical University, Nanning, China; University of Navarra, SPAIN

## Abstract

**Background:**

Despite the identification of many signaling pathways involved in colorectal cancer (CRC) tumorigenesis, metastatic CRC still remains one of the major causes of cancer related death. NIK and IKKβ-binding protein (NIBP) is one of the key regulators of the NF-κB signaling pathway, which has been implicated in CRC metastasis. The aim of this study was to investigate the possible role of NIBP in CRC metastasis through its regulation of NF-κΒ and extracellular regulated kinase/c-Janus kinase (ERK/JNK) signaling pathways.

**Methods:**

In this study NIBP, phosphorylated (p)-p65, p-ERK1/2, and p-JNK1/2 expression was examined in 130 CRC, and 25 adenoma tissue samples were studied by immunohistochemistry. NIBP shRNA knockdown was performed in HCT116 cells, and NF-κB and ERK/JNK pathway activity was measured after TNF-α stimulation *in vitro* and *in vivo*.

**Results:**

We found that NIBP, p-p65, p-ERK1/2, and p-JNK1/2 expression was higher in late stages of CRC compared to early stages or adenomas. Expression of p-p65, p-IκBα, p-IκBβ, p-ERK1/2, and p-JNK1/2 was inhibited in TNF-α stimulated HCT116 cells following NIBP knockdown. Nevertheless, p-ERK1/2 expression in un-transfected and NIBP knockdown HCT116 cells remained the same in the absence of TNF-α stimulation. Furthermore, cell motility and invasion were reduced in HCT116 cells following NIBP knockdown even after TNF-α treatment. Finally, primary tumor weight and liver metastasis were reduced in nude mice with orthotopically transplanted NIBP knockdown of HCT116 cells.

**Conclusion:**

In conclusion, we demonstrated that NIBP knockdown reduces colorectal cancer metastasis through down-regulation of canonical NF-κΒ signaling and suppression of ERK and JNK signaling.

## Introduction

Colorectal cancer (CRC) is one of the most common types of cancer with over 130,000 newly diagnosed cases in the United States annually. The treatment options for metastatic colorectal cancer (mCRC) are limited, making mCRC a significant clinical challenge[1]. Many signaling pathways and molecules involved in the development and progression of CRC have been identified; however, which molecules are specifically involved in regulating metastasis still remain to be clarified[2]. Therefore, research examining the molecular processes that govern CRC metastasis may provide new targets for the treatment of mCRC.

The transcription nuclear factor κB (NF-κΒ) signaling pathway, which has a pivotal role in tumorigenesis, is activated in response to cytokines, growth factors, oncoproteins, and stress signals, and can follow two distinct activation pathways[2]. In the canonical pathway, NF-κΒ is triggered by tumor necrosis factor-α (TNF-α) and interleukin (IL)-1β, and is dependent on the inhibitor of NF-κB kinase (IκB or IKK). Under basal conditions NF-κΒ binds to IκB in the cytoplasm and, following proteasomal degradation of IκB, NF-κB translocates to the nucleus where it facilitates gene transcription. As a relatively novel regulator of canonical NF-κB signaling, NIK and IKKβ-binding protein (NIBP) plays a dual role as an activator of NF-κB through its direct interactions with NIK and IKKβ[3]. NIBP enhances cytokine-induced NF-κB activation through potentiating IKKβ kinase activity and also has a role in protein trafficking[3]. High NIBP expression has been reported in cancer cell lines and tumor tissues[4]. Knockdown of NIBP has been shown to reduce TNF-α induced NF-κB activation, which may prevent cell invasion and differentiation. In our previous study we showed that NIBP overexpression promoted invasion of colorectal cancer cells through activation of matrix metalloproteinases (MMPs)[5]. In addition, it has been shown that NIBP knockdown inhibits HCT116 colon cell proliferation, invasion, and tumor formation, while NIBP overexpression promotes these processes[4]. NIBP has also been implicated in trans-Golgi network and antiviral defense[6, 7].

Mitogen activated protein kinase (MAPK) signaling pathways, mediated through extracellular regulated kinase (ERK) and c-Jun N terminal kinase (JNK), represent other important regulatory networks involved in tumorigenesis, including regulation of proliferation and apoptosis[8]. Recent studies have shown that MAPKs are involved in NF-κB activation. Indeed, ERK expression was up-regulated by NF-κB and activating transcription factor 3 (ATF3) activation, which was followed by an increase in apoptosis in human colorectal cancer cells[9, 10]. In contrast, NF-κB activation was reduced through inhibition of the intracellular JNK signaling cascade[9]. Thus, TNF-α not only activated the canonical NF-κB pathway, but also increased JNK activity[11]. However, the link between NF-κB and MAPKs is still unclear.

As a key regulator of canonical NF-κB signaling, the mechanisms of NIBP regulated NF-κB activation and possible interactions with the ERK and JNK signaling pathways in mCRC have not yet been established. Therefore, the aim of this study was to examine the possible association of NIBP with these signaling pathways in patients with colorectal adenomas and adenocarcinomas. In addition, the consequences of NIBP knockdown were explored in a human adenocarcinoma HCT116 cell model.

## Materials and Methods

### Patients and tissue specimens

A total of 130 consecutive patients with sporadic colorectal cancer scheduled to undergo curative resection and 25 patients with colorectal adenoma diagnosed by colon endoscopy from March 2013 to October 2013 at the First Hospital of Guangxi Medical University (Nanning, China) were included in this study. Tumor tissue samples obtained at the time of surgery were fixed and embedded in paraffin. Cancer staging was determined by imaging studies and operative findings with histological diagnosis according to the NCCN Clinical Practice Guidelines in Oncology for Colon Cancer (Version 3, 2013). All patients provided written informed consent, and the study protocol was approved by the Institutional Review Board for Human Genetic and Genomic Research, in accordance with the Declaration of Helsinki.

### Antibodies and reagents

Antibodies against p65 (cat.no. 4764), phospho-p65 (p-p65, cat.no. 3033), IκBα (cat.no. 8635), phospho-IκBα (p-IκBα, cat.no. 2859), IκBβ (cat.no. 8635), phospho-IκBβ (p-IκBβ, cat.no. 4921), ERK1/2 (cat.no. 4695), phospho-ERK1/2 (p-ERK1/2, cat.no. 4370), JNK (cat.no. 9252), phospho-JNK (p-JNK1/2, cat.no. 4668) and glyceraldehyde phosphate dehydrogenase (GAPDH) (cat.no. 2118) were purchased from Cell Signaling Technology (Beverly, USA). Antibodies against NIBP (cat.no. 16014-1-AP) were obtained from Proteintech Group (Rosemont, USA). PVDF membrane (cat.no. 162–0181) was obtained from Bio-Rad Laboratories (Hercules, USA). Pierce ECL Western Blotting Substrate (cat.no. 32209) was obtained from Thermo Fisher Scientific (Waltham, USA). Blasticidin S (cat.no. 203351) was purchased from EMD Millipore (Darmstadt, Germany). TNF-α (cat.no. T6674) and Matrigel (cat.no. E1270) were purchased from Sigma-Aldrich Co. (St. Louis, USA). The 24-well transwell plates (cat.no.3422) were obtained from Corning Inc. (Corning, USA).

### Cell culture and transfection

The human adenocarcinoma cell line HCT116 obtained from American Type Culture Collection (ATCC) was used for stable NIBP knockdown transfections. In brief, HCT116 cells were cultured in high glucose Dulbecco's modified Eagle's medium (DMEM) supplemented with 10% fetal bovine serum (FBS) and penicillin/streptomycin (100 U/ml). The NIBP short hairpin RNA (shRNA) (3’-GGAAGCTGTCCTGAATTTCAA-5’) was cloned into a pcDNA6.2-EGFP-NIBP-miR vector and introduced into HCT116 cells via lentiviral transfection. Cells carrying the NIBP shRNA and empty vector (NC) were selected by culturing in the presence of 5 μg/ml blasticidin S (Darmstadt, Germany) for more than two weeks. Stable clones were verified by Western blot and one stably transfected NIBP knockdown HCT116 cell clone was chosen for subsequent experiments. To determine the effect of NIBP on the activation of the NF-κΒ canonical pathway, HCT116 cells were incubated with 20 ng/ml TNF-α for 48 h in NIBP knockdown and control un-transfected cells.

### Western blot analysis

For Western blot analysis cells were lysed in Triton X-100-based lysis buffer. Protein concentration in the supernatant was determined using Bradford colorimetry. Next, 40 μg of protein from each sample were denatured and separated by SDS-PAGE and electro blotted onto PVDF membrane. Following blocking in 5% non-fat milk in Tris buffered saline with Tween (TBST) for 1 h, membranes were incubated overnight at 4°C with appropriate antibodies as follows: NIBP (1:100), p65 (1:500), p-p65 (1:500), IκBα (1:500), p-IκBα (1:500), IκBβ (1:500), p-IκBβ (1:500), ERK1/2 (1:500), p-ERK1/2 (1:500), JNK1/2 (1:500), p-JNK1/2 (1:500) and GAPDH (1:5,000). After washing with phosphate buffered saline (PBS), PVDF membranes were incubated with horseradish peroxidase (HRP)-conjugated secondary antibody (1:3,000) for 1 h. Protein signals were visualized using enhanced chemiluminescence reagents, according to the manufacturer’s instructions.

### Immunohistochemistry

All tissue samples were fixed with 4% paraformaldehyde for 12 h, embedded in paraffin and cut into 5 μm sections. Next, sections were deparaffinized, rehydrated, and endogenous peroxidases were blocked in methanol with 30% H_2_O_2_. After antigen retrieval induced by heat, sections were blocked in 10% normal goat serum in PBS for 1 h, and incubated with primary antibody for 4 h. Next, HRP-conjugated secondary antibody was applied for 30 min. Immunohistochemical reaction was visualized using DAB chromogen. All slides were evaluated by two pathologists. Evaluation of the nuclear staining reaction was performed in accordance with the immunoreactive score (IRS) proposed by Remmele and Stegner[12]: IRS = SI (staining intensity) x PP (percentage of positive cells). SI was defined as 0, negative; 1, weak; 2, moderate; and 3, strong. PP was defined as 0, no positive cells present; 1, 10% positive cells; 2, 11–50% positive cells; 3, 51–80% positive cells; and 4, more than 80% positive cells. Ten visual fields from different areas of each tumor were used for IRS evaluation. Tumor slides with at least 3 IRS points in our study were classified as immunoreactive.

### Invasion and motility assays

To prepare transwell plates for invasion assay, inserts were coated with Matrigel, which was first thawed at 4°C overnight and then diluted at a concentration of 5 mg/ml in cold serum-free DMEM. Next, 100 μl of the diluted Matrigel were poured into the upper chamber of the 24-well transwell insert and incubated at 37°C for at least 4 h for solidification. Next, HCT116 cells were grown to approximately 80% confluence in cell culture flasks and harvested by trypsinization, washed three times with DMEM containing 1% FBS, and resuspended at a density of 5 x 10^5^ cells/ml. Next, 1 x 10^5^ cells were added onto the transwell insert and the lower chamber of the transwell was filled with 600 μl of DMEM containing 20% FBS. Cells were then incubated at 37°C for 24 h and the non-invading cells on the top of the transwell insert were scraped off with a cotton swab. Inserts were then removed from plates, stained with crystal violet solution, and the optical density (OD) was read at 590 nm. Similarly, the tumor cell motility assay was performed using 24-well transwell plates with uncoated inserts in the same manner as the invasion assay. The data are presented as mean ± SD of three independent experiments.

### Orthotopic transplantation model of human colonic tumor

For *in vivo* experiments, un-transfected or NIBP knockdown HCT116 cells (7 x 10^6^ cells) were inoculated subcutaneously into the dorsal surfaces of BALb/c nude male mice obtained from SLAC Laboratory Animal Center (Shanghai, China). When xenografts of approximately 500 mm^3^ were established, they were excised and divided into 1 mm^3^ pieces. Two of these pieces were then orthotopically implanted into the colons of other male BALb/c nude mice as previously reported[13]. Briefly, for operative procedures, animals were anesthetized with isoflurane inhalation. A 1 cm laparotomy was performed, and both the cecum and ascending colon were exteriorized. Using 7 X magnification and microsurgical techniques, the serosa was disrupted in two different locations. Xenografts were subserosally implanted using a nylon suture at the two points of serosal disruption. The bowel was then returned to the peritoneal cavity and the abdomen was closed with interrupted vicryl sutures[14]. Each mouse in the experimental group was observed for up to 6 weeks, and mice were weighed every week. After 4 weeks, mice were sacrificed and primary tumor, metastatic tumor, and serum were snap-frozen in liquid nitrogen for subsequent analyses. All animals were checked on daily basis to monitor their health. All of the mice used in this study were euthanized by cervical dislocation. All animal care and studies were conducted in accordance with the Medical ethics committee of the First Affiliated Hospital of Guangxi Medical University for Ethical Approval for Research Involving Animals (Nanning, China, permit number: KY-036).

### Statistical analysis

All data are presented as mean ± standard deviation (SD). The statistical significance of differences between the means was evaluated using the unpaired Student's t test or the one-way analysis of variance (ANOVA) test. Statistical analysis was performed using the Statistical Package for the Social Sciences (SPSS) 20.0. *p* < 0.05 was considered significant.

## Results

### Clinical characteristics of CRC patients

A total of 25 patients with colorectal adenomas and 130 patients with colorectal adenocarcinomas were included in the study. The group of colorectal patients consisted of 103 males and 52 females. The age of patients ranged from 25 to 83 years old at first diagnosis. According to the NCCN CRC classification, 22 patients were at stage I, 53 were at stage II, 33 were at stage III, and 22 were at stage IV (Table 1). Seventy-nine tumors were located in the left-sided colon (descending and sigmoid colon and rectum), and 51 tumors were located in the right-sided colorectum (caecum, ascending, and transverse colon up to the splenic flexure). Twenty-six tumors were mucinous adenocarcinoma and 104 were tubular adenocarcinoma as identified by pathologists. The maximum diameter was less than 2 cm in 10 tumors, between 2 and 5 cm in 67 tumors, and larger than 5 cm in 53 tumors. Eighteen CRCs were highly differentiated, 88 were moderately differentiated, and 24 were low differentiation.

**Table 1:** CRC patient clinicopathological characteristics and IRS values for NIBP, p-p65, p-ERK1/2, and p-JNK1/2 immunohistochemical expression.

	N	NIBP IRS	p-p65 IRS	p-ERK1/2 IRS	p-JNK1/2 IRS
Total					
adenoma	25	1.24±0.61	1.48±1.92	1.30±0.87	0.87±0.57
TNM I	22	1.97±1.17	2.69±2.00	2.00±1.82	1.50±1.03
TNM II	53	2.49±1.21^a^	3.06±2.36^a^	2.81±1.68^a^	2.78±1.14^a^
TNM III	33	5.63±2.70^abc^	6.29±3.72^abc^	6.24±1.12^abc^	6.18±1.04^abc^
TNM IV	22	7.15±2.69^abc^	9.10±2.81^abcd^	7.78±1.70^abcd^	7.95±1.50^abcd^
Pathological type					
adenoma	25	1.24±0.61	1.48±1.92	1.87±0.87	0.87±0.57
mucinous adenocarcinoma	26	3.66±2.85^e^	5.06±3.73^e^	4.24±2.77^e^	4.38±2.81^e^
tubular adenocarcinoma	104	4.07±2.79^e^	4.78±3.65^e^	4.42±2.70^e^	4.28±2.57^e^
CRC location					
left-sided colorectum	79	4.00±2.75	4.90±3.65	4.31±2.70	4.12±2.62
right-sided colon	51	3.97±2.89	4.70±3.69	4.50±2.73	4.58±2.58
Maximum diameter of CRC					
<2 cm	10	2.06±1.24	2.38±1.91	2.56±2.41	2.76±2.28
2–5 cm	67	4.08±2.66^f^	4.93±3.74^f^	4.62±2.63^f^	4.39±2.55
>5 cm	53	4.23±3.06^f^	5.18±3.67^f^	4.43±2.76^f^	4.47±2.68
CRC histologic differentiation					
High differentiation	18	3.32±2.39	3.40±2.58	3.69±2.21	2.66±1.72
Moderate differentiation	88	3.90±2.78	4.94±3.72	4.23±2.80^g^	4.27±1.63^g^
Low differentiation	24	4.83±3.04	5.54±3.92	5.49±2.44^g^	5.63±2.37^g^

a *vs* adenoma, *p* < 0.05; b *vs* TNM I, *p* < 0.05; c *vs* TNM II, *p* < 0.05; d *vs* TNM III, *p* < 0.05; e *vs* adenoma, *p* < 0.05; f *vs* < 2 cm CRC, *p* < 0.05; g *vs* high differentiation *p* < 0.05.

### NIBP, p-p65, p-ERK, and p-JNK expression in colorectal adenomas and adenocarcinomas

**Fig 1:**
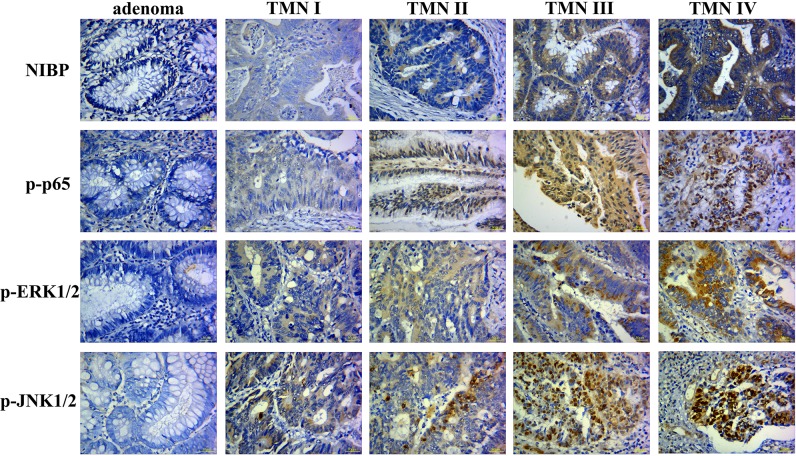
Immunohistochemical analysis of NIBP, p-p65, p-ERK1/2, and p-JNK1/2 expression in colorectal adenomas and adenocarcinomas. NIBP, p-p65, p-ERK1/2, and p-JNK1/2 protein expression was higher in late CRC stages (TNM III and TNM IV) compared to early CRC stages (TNM I and TNM II) or adenomas. In addition, NIBP, p-p65, p-ERK1/2, and p-JNK1/2 expression was higher in mucinous adenocarcinomas and tubular adenocarcinomas compared to adenomas.

In this study, we used immunohistochemistry to assess NIBP, p-p65, p-ERK1/2, and p-JNK1/2 expression in patients with adenomas and sporadic adenocarcinomas (Fig 1). The IRS values for NIBP, p-p65, p-ERK1/2, and p-JNK1/2 were higher in late CRC stages (TNM III and TNM IV) compared to early stage cancers and adenomas *p* < 0.05, Table 1). In addition, IRS values for NIBP, p-p65, p-ERK1/2, and p-JNK1/2 were higher in mucinous adenocarcinomas and tubular adenocarcinomas compared to adenomas. The IRS values for NIBP were lower in smaller tumors that had maximum diameters less than 2 cm (*p* < 0.05, Table 1). The IRS values for p-ERK1/2 and p-JNK1/2 were lower in highly differentiated tumors when compared to moderately and low differentiated tumors (*p* < 0.05, Table 1). However, we did not observe any differences in the IRS for NIBP and p-p65.

### NIBP knockdown inhibits activation of the NF-κΒ canonical and ERK/JNK pathways in HCT116 cells in vitro

**Fig 2:**
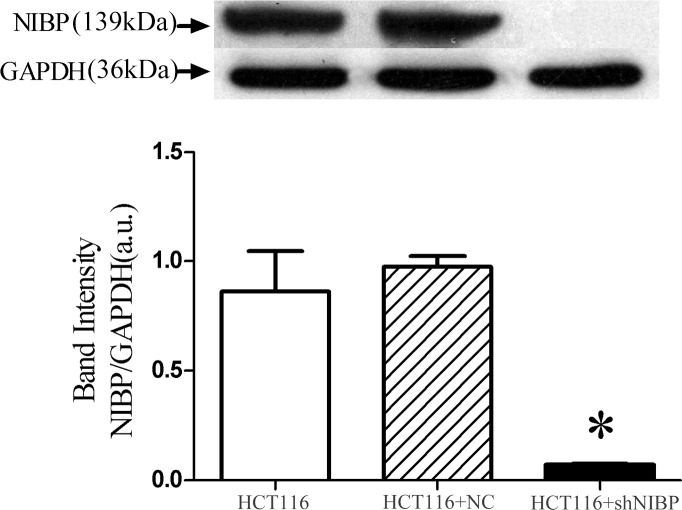
Lentivirus-mediated NIBP knockdown in HCT116 cells. Control un-transfected HCT116 cells had higher NIBP protein expression compared to NIBP-knockdown HCT116 cells. Cells transfected with an empty vector (NC) had high NIBP expression, similar to the control un-transfected HCT116 cells, as determined by Western blot analysis. * *vs*. un-transfected HCT116, *p* < 0.05.

In our study the un-transfected control HCT116 cells showed high NIBP protein expression[4]. Stable NIBP knockdown in HCT116 cells resulted in low NIBP expression, while cells transfected with an empty vector (NC) had high NIBP protein expression similar to the control un-transfected HCT116 cells (Fig 2).

**Fig 3:**
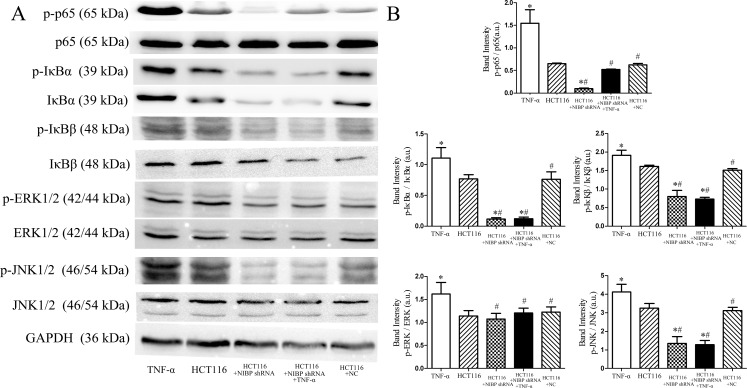
NIBP knockdown inhibits activation of the canonical NF-κΒ and ERK and JNK mediated pathways in HCT116 cells. A. Representative results of Western blot analysis of control un-transfectedand NC transfected HCT116 cells, and NIBP knockdown HCT116 cells with or without TNF-αtreatment. B. p-p65, p-IκBα and p-IκBβ expression was highest in control un-transfected HCT116 cells after TNF-α treatment; and TNF-α treatment reduced p-p65 levels similar to total p65 in NIBP knockdown HCT116 cells. Additionally, p-ERK1/2 expression was up-regulated in TNF-α treated control un-transfected HCT116 cells, and this increase was reduced by NIBP knockdown; however, no difference was observed in p-ERK1/2 expression between the control un-transfected cells and NIBP knockdown cells without TNF-α treatment. Moreover, p-JNK1/2 expression was increased in control un-transfected cells treated with TNF-α and decreased in knockdown NIBP cells, irrespective of whether they were treated with TNF-α or not. * *vs*. un-transfected HCT116, *p* < 0.05; # *vs*. TNF-α treatment, *p* < 0.05.

In order to examine the influence of NIBP on canonical NF-κΒ pathway activation, HCT116 cells (NC and NIBP shRNA) were incubated with 20 ng/ml TNF-α for 48 h. TNF-α treatment increased protein expression of p65, IκBα, IκBβ, p-p65, p-IκBα and p-IκBβ in NC HCT116 cells. Contrary to these findings, expression of these proteins was significantly lower in NIBP shRNA transfected HCT116 cells regardless of whether they were treated with TNF-α or not (*p* < 0.05; Fig 3).

In control un-transfected HCT116 cells TNF-α treatment induced phosphorylation of ERK1/2 and JNK1/2. Contrary to these findings, phosphorylation of JNK1/2 was inhibited in NIBP shRNA HCT116 cells (*p* < 0.05; Fig 3); nevertheless, phosphorylation of ERK1/2 was not affected (*p* > 0.05; Fig 3). However, when NIBP shRNA transfected HCT116 cells were treated with TNF-α the phosphorylation of ERK1/2 and JNK1/2 was reduced (*p* < 0.05; Fig 3). Collectively, these results indicate that NIBP knockdown inhibits activation of the NF-κΒ canonical pathway by decreasing phosphorylation of p65, IκBβ, and IκBβ in HCT116 cells, ultimately reducing the effects of TNF-α stimulation. However, NIBP knockdown inhibited the ERK/JNK pathway and, in part, reduced phosphorylation of ERK1/2 and JNK1/2 after the TNF-α treatment.

### NIBP knockdown decreases HCT116 cell motility and invasion in vitro

**Fig 4:**
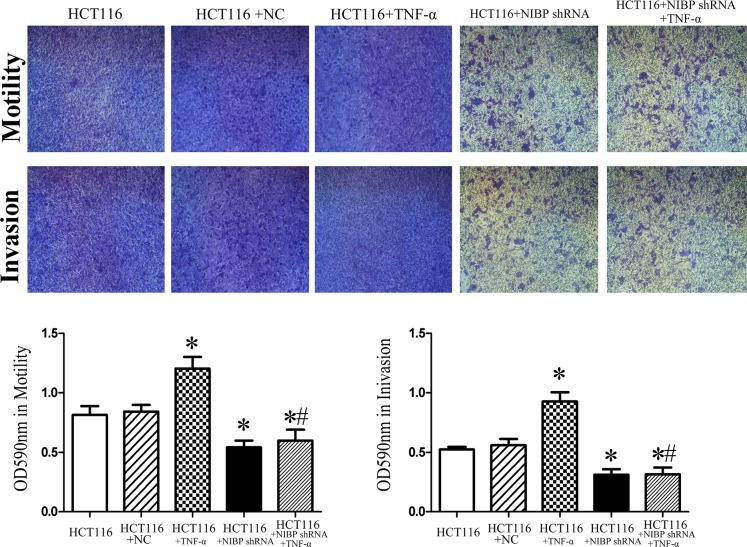
NIBP knockdown increases cell motility and invasion in HCT116 cells *in vitro*. Motility and invasion capability of control un-transfected and NC transfected HCT116 cells as well as NIBP knockdown HCT116 cells with or without TNF-α treatment. OD at 590 nm for motility and invasion assays was the highest in control un-transfected HCT116 cells treated with TNF-α. In NIBP knockdown HCT116 cells motility and invasion were low regardless of TNF-α treatment. * *vs*. un-transfected HCT116, *p* < 0.05; # *vs*. TNF-α treatment, *p* < 0.05.

Cell motility and invasion are essential for metastatic spread of tumors, both locally as well as at distant sites in the organism. Therefore, we examined the influence of NIBP knockdown on these two important cellular processes. In our study NIBP shRNA knockdown reduced cell motility and invasion of HCT116 cells, even after cells were treated with TNF-α (*p* < 0.05; Fig 4). These results further support the hypothesis that NIBP knockdown inhibits activation of the canonical NF-κΒ and ERK/JNK pathways.

### NIBP knockdown decreases liver metastases and tumor proliferation of HCT116 cells in vivo

**Fig 5:**
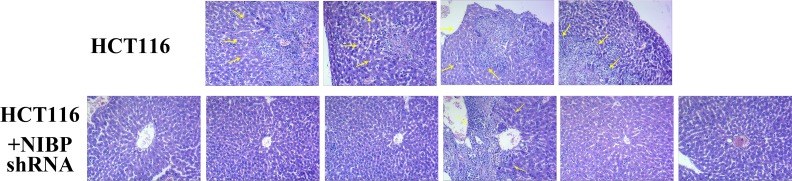
Liver metastasis of HCT116 orthotopic transplantation in the nude mice model. Liver metastasis of control un-transfected HCT116 cells and NIBP knockdown cells in the orthotopic transplantation model. The liver metastatic potential of HCT116 cells was decreased by NIBP knockdown, as indicated by the yellow arrows.

**Fig 6:**
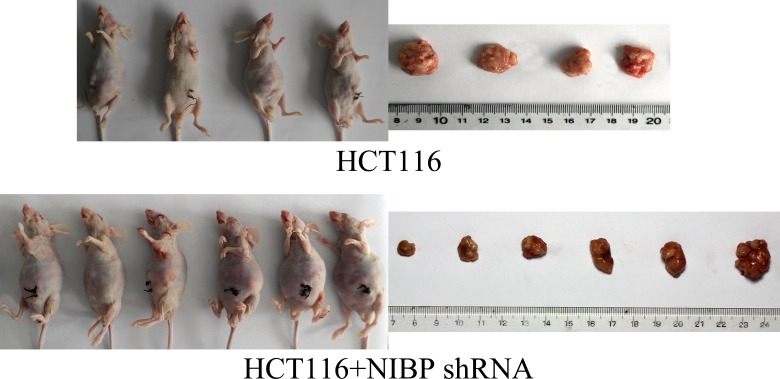
HCT116 orthotopic transplantation in the nude mice model. Orthotopic transplantation of control un-transfected HCT116 cells and NIBP knockdown cells in nude mice. Primary tumors weighed less in mice transplanted with NIBP knockdown HCT116 cells compared to mice transplanted with control un-transfected HCT116 cells (967 ± 515 mg *vs*. 1738 ± 396 mg; *p* = 0.036).

In this study, the metastatic capability of un-transfected as well as NIBP shRNA transfected HCT116 cells was examined following colonic orthotopic implantation of subcutaneously grown xenografts. The control un-transfected HCT116 cells had more metastatic potential than NIBP knockdown HCT116 cells. Implanted un-transfected HCT116 cells resulted in cell metastasis to the liver in all four mice. The metastatic potential of HCT116 cells was decreased by NIBP knockdown, and only one of six mice had liver metastasis (Fig 5, Table 2). Additionally, primary tumors weighed less in mice implanted with NIBP knockdown HCT116 cells compared to mice with control un-transfected HCT116 cells (967 ± 515 mg *vs*.1738 ± 396 mg; *p* = 0.036; Fig 6). Collectively, these results indicate that NIBP knockdown in HCT116 cells decreases their metastatic potential.

**Table 2:** NIBP knockdown inhibited the formation of HCT116 liver metastases in *in vivo*.

animal group	Total (n)	liver metastases (n)		*p*
		+	-	
NIBP knockdown HCT116	6	1	5	0.048
un-transfected HCT116 e	4	4	0	

## Discussion

Colorectal cancer is a major public health issue with regards to malignant diseases[15]. Despite the current knowledge about the molecular pathology of CRC, the 5-year relative survival rate of patients, especially those in late stage mCRC, is still low [16–18]. The role of NF-κB signaling in CRC has been explored in recent years and several mechanisms have been proposed to explain the regulation of persistent NF-κB activation in tumorigenesis[18]. In brief, it has been shown that NF-κB promotes CRC invasiveness by increasing vascular endothelial growth factor (VEGF), intracellular cell adhesion molecule (ICAM), vascular cell adhesion molecule (VCAM), and MMP expression[16]. Jeong *et*. *al*., showed that the ERK pathway was activated by reactive oxygen species (ROS) generation, and the NF-κB pathway reduced apoptosis in human colorectal cancer cells[10]. In addition, TNF-α activated both the canonical NF-κB and JNK pathways and increased expression of pro-inflammatory factors[11]. In our previous study we showed that HT-29 cell invasiveness was enhanced via activation of ERK1/2 and MMP-2/9[19]. In this study phosphorylation of p65, ERK1/2, and JNK1/2 was increased in patients with late CRC TNM stages. Furthermore, phosphorylation of p65, IκBα, IκBβ, ERK1/2, and JNK1/2, was increased in HCT116 cells treated with TNF-α. Moreover, cell motility and invasion properties were enhanced in TNF-α treated HCT116. Based on these findings, it can be concluded that both the canonical NF-κB and the ERK and JNK pathways increase with CRC progression and play important roles in cancer metastasis. This is not surprising since, in addition to the known activation of NF-κB signaling, activation of the ERK and JNK mediated pathways has also been reported in the pathogenesis, progression, and oncogenic behavior of human colorectal cancer[18, 19].

It has been previously reported that canonical NF-κB pathway-dependent gene expression is increased when NIBP expression is up-regulated[20]. NIBP is highly expressed in breast cancer and colorectal cancer while its expression is low in immune organs in which NF-κB is known to perform important biological functions[3, 4]. In addition, it has been shown that NIBP expression correlates with tumorigenesis in CRC[21]. Some studies have reported crosstalk between NF-κB and MAPK cascades[22–24], with one of the most important mediators of these interactions being the growth arrest and DNA damage-inducible 45 (Gadd45) family[25]. However, the exact molecular mechanism underlying this crosstalk still remains unknown. In this study, phosphorylation of p65, ERK1/2 and JNK1/2 increased in late CRC stages, as did the expression of NIBP. Based on these results we hypothesized that NIBP regulates the metastatic potential of tumor cells through induction of the canonical NF-κB and ERK and JNK pathways. In order to test this hypothesis, we decreased NIBP expression in the human adenocarcinoma cell line HCT116, which is known to have high invasive capability. NIBP knockdown in HCT116 cells decreased phosphorylation of p65, IκBα, IκBβ, and JNK1/2 and attenuated *in vitro* motility and invasion. In addition, NIBP knockdown inhibited the TNF-α mediated activation of the canonical NF-κB and ERK and JNK pathways. NIBP knockdown also inhibited the metastatic potential of HCT116 cells in nude mice. These data indicate that knockdown of NIBP reduces metastatic potential of CRCs through inhibition of the canonical NF-κB pathway and suppression of ERK and JNK mediated signaling.

In conclusion, we have shown that knockdown of NIBP reduces colorectal cancer metastasis through down-regulation of the canonical NF-κΒ signaling pathway, as well as via suppression of MAPK signaling mediated through ERK and JNK. These findings might prove useful in establishing new targets for anticancer therapy especially for advanced stages of CRC for which current treatment options are still limited.
